# Spondylodiscitis After Surgery for Pelvic Organ Prolapse: Description of a Rare Complication and Systematic Review of the Literature

**DOI:** 10.3389/fsurg.2021.741311

**Published:** 2021-10-29

**Authors:** Guglielmo Stabile, Federico Romano, Ghergana A. Topouzova, Francesco Paolo Mangino, Giovanni Di Lorenzo, Antonio Simone Laganà, Nicolò De Manzini, Giuseppe Ricci

**Affiliations:** ^1^Department of Gynecology and Obstetrics, Institute for Maternal and Child Health Istituto di Ricovero e Cura a Carattere Scientifico (IRCCS) “Burlo Garofolo”, Trieste, Italy; ^2^University Clinical Department of Medical, Surgical and Health Sciences, University of Trieste, Trieste, Italy; ^3^Department of Obstetrics and Gynecology, ‘Filippo Del Ponte' Hospital, University of Insubria, Varese, Italy

**Keywords:** spondylodiscitis, sacrocolpopexy, rectopexy, prolapse, mesh

## Abstract

**Background:** Spondylodiscitis can be a rare complication of gynecological surgery, typically of procedures involving the sacrum and the sacrospinous ligament. This report presents a case of spondylodiscitis arising after a laparoscopic sacrocolpopexy with a mesh. We also review the literature finding 52 cases of spondylodiscitis following sacrocolpopexy and (or) rectopexy with or without a mesh.

**Methods:** We performed a comprehensive search from the electronic databases MEDLINE (Pubmed), Scopus, Web of Science, Embase, CINAHL, and Google Scholar from 1990 to February 2021 in order to identify case reports or case series reporting on spondylodiscitis after rectopexy or sacrocolpopexy.

**Results:** We identified 52 total postoperative spondylodiscitis. We examined the mean age of patients, the surgical history, the time from initial surgery to spondylodiscitis, the presenting symptoms, the diagnostic tools, the medical and surgical treatment, the type of mesh used, the surgical access, and the possible causes of spondylodiscitis.

**Conclusions:** Diagnosis of spondylodiscitis may be challenging. From our review emerges that recurrent pelvic pain and lumbosciatalgia may be signals of lumbar spondylodiscitis. Magnetic resonance is the gold standard examination for spondylodiscitis. Surgical practice needs to be improved further in order to establish the best procedure to minimize the incidence of this complication. Awareness of symptoms, timely diagnosis, and treatment are fundamental to prevent irreversible complications.

## Introduction

Pelvic organ prolapse results from laxities of the ligaments, fascia, and muscles supporting the pelvic organs (1). Rectopexy and sacrocolpopexy are established surgical techniques to restore anatomy and organ function. The promontory of the sacrum is widely used as the proximal fixation point for laparotomic or laparoscopic- or robotic-assisted recto- and sacrocolpopexy as well for other surgical techniques (2). Depending on the technique, the organ fixation is performed either by direct sutures or by using a mesh that is sutured or tacked to the promontory of the sacrum. We report a case of spondylodiscitis arising as a complication of a laparoscopic sacrocolpopexy with a mesh. The spondylodiscitis had not been recognized immediately and the diagnosis was reached only after a magnetic resonance was performed for the recurrence of pelvic pain and lumbosciatalgia. The report also discusses 52 cases available in literature of spondylodiscitis following sacrocolpopexy and (or) rectopexy with or without a mesh. We evaluate the current knowledge for the diagnosis and management of spondylodiscitis after surgery.

## Case

**Figure 1:**
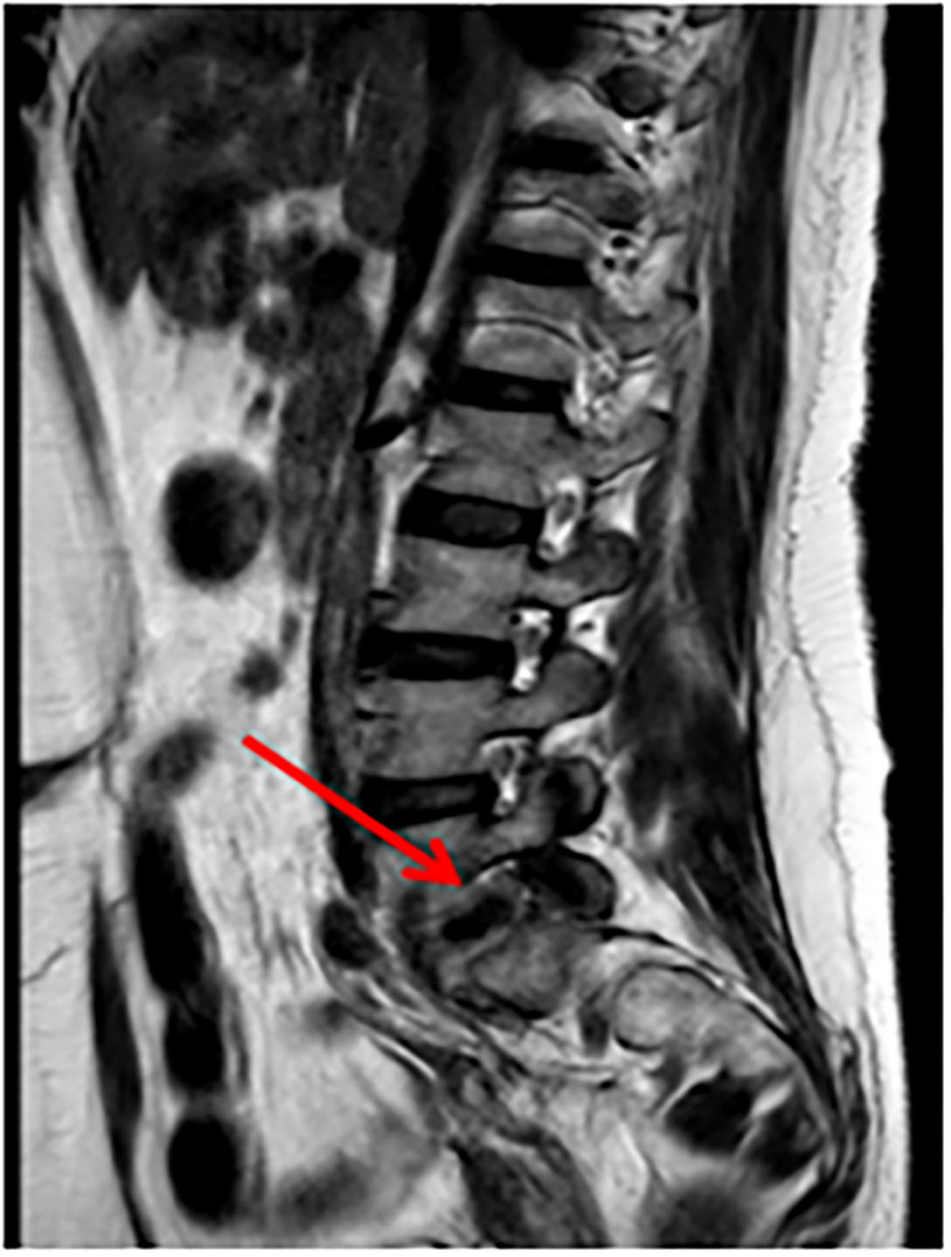
Pelvic MRI: enhancement of soft tissues surrounding the L5-S1 vertebrae (arrow). MRI, magnetic resonance imaging.

**Figure 2:**
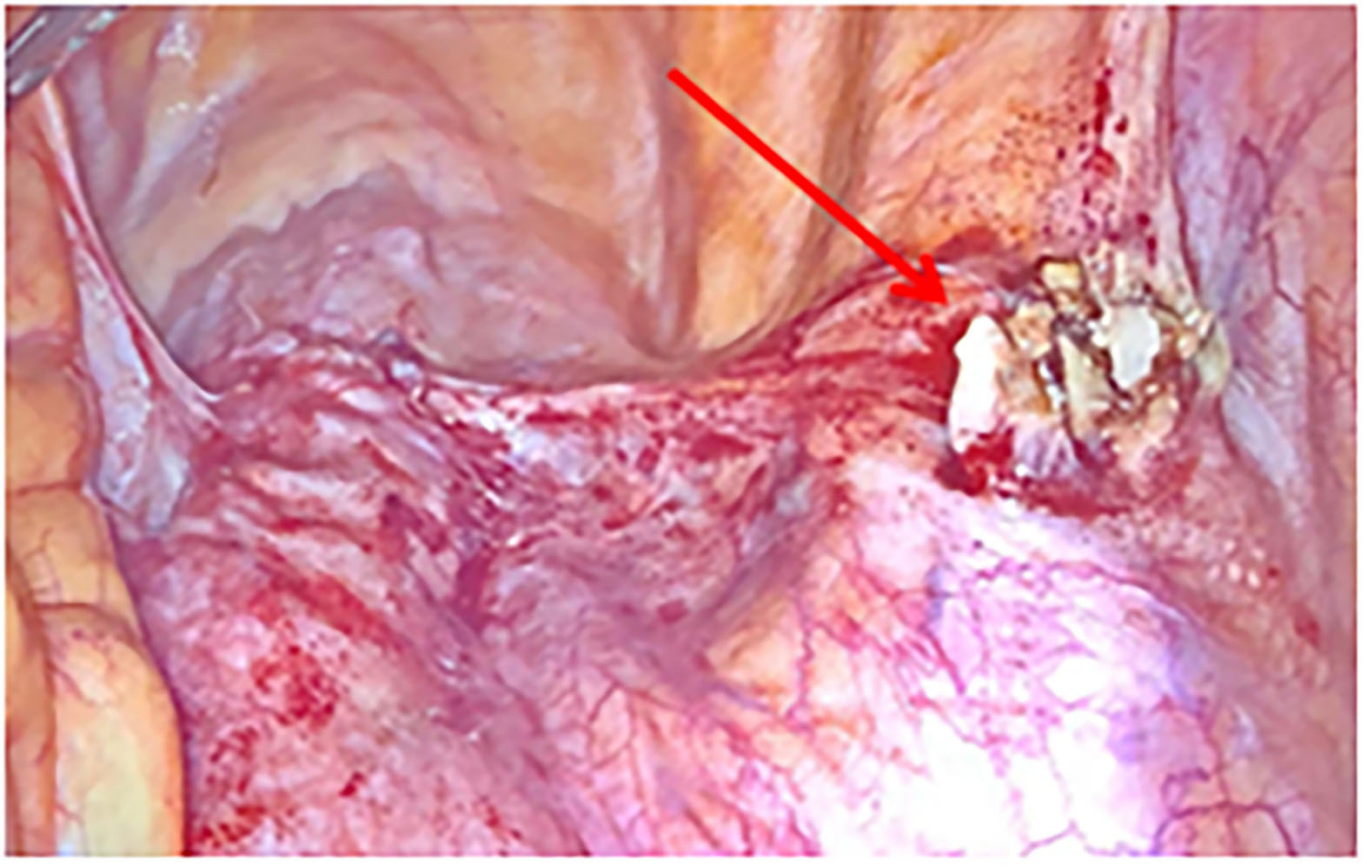
Laparoscopic view: purulent collection involving the polypropylene mesh (arrow).

**Figure 3:**
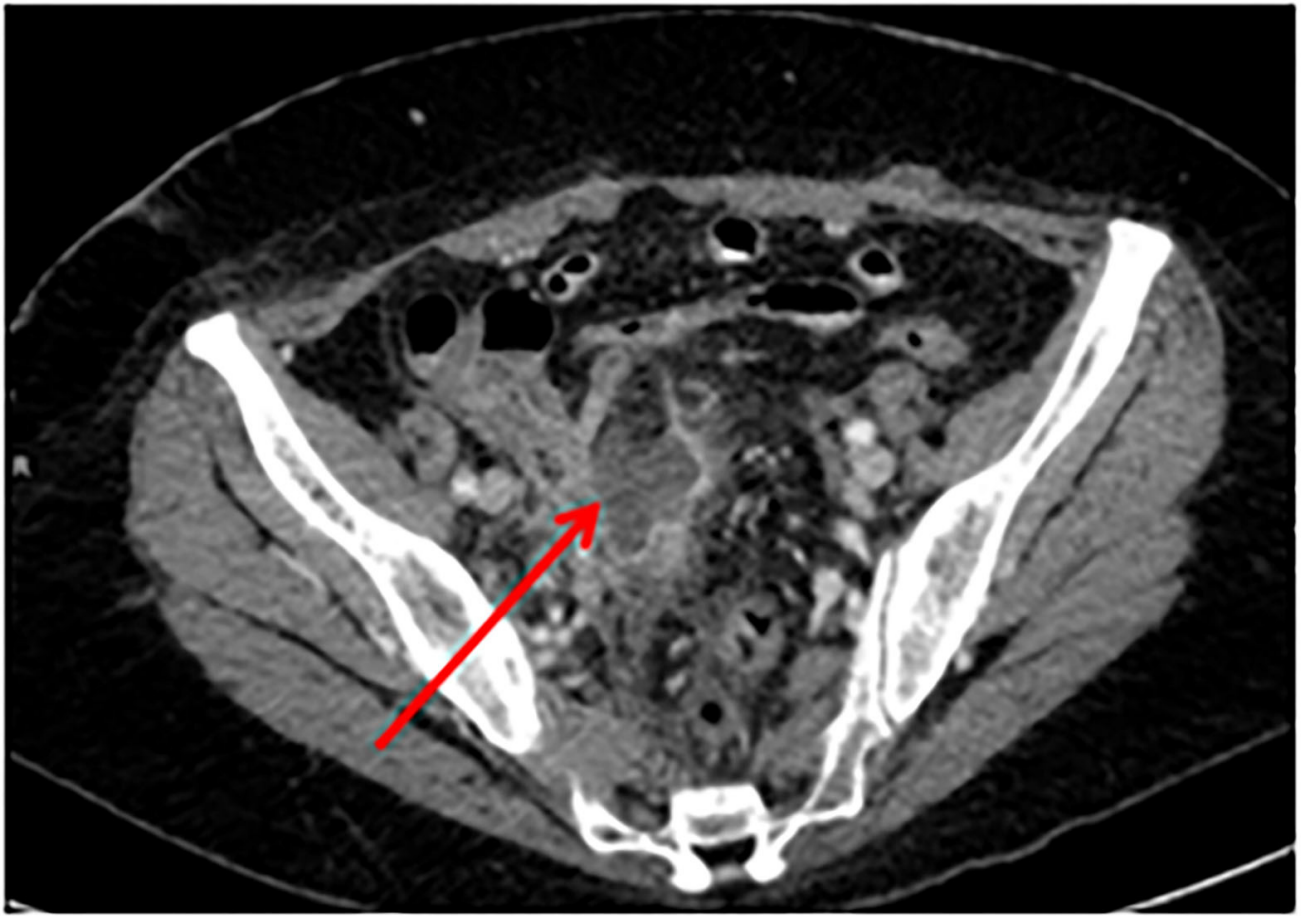
CT: purulent collection of the pelvis between the vagina and the rectum (arrow). CT, computed tomography.

**Figure 4:**
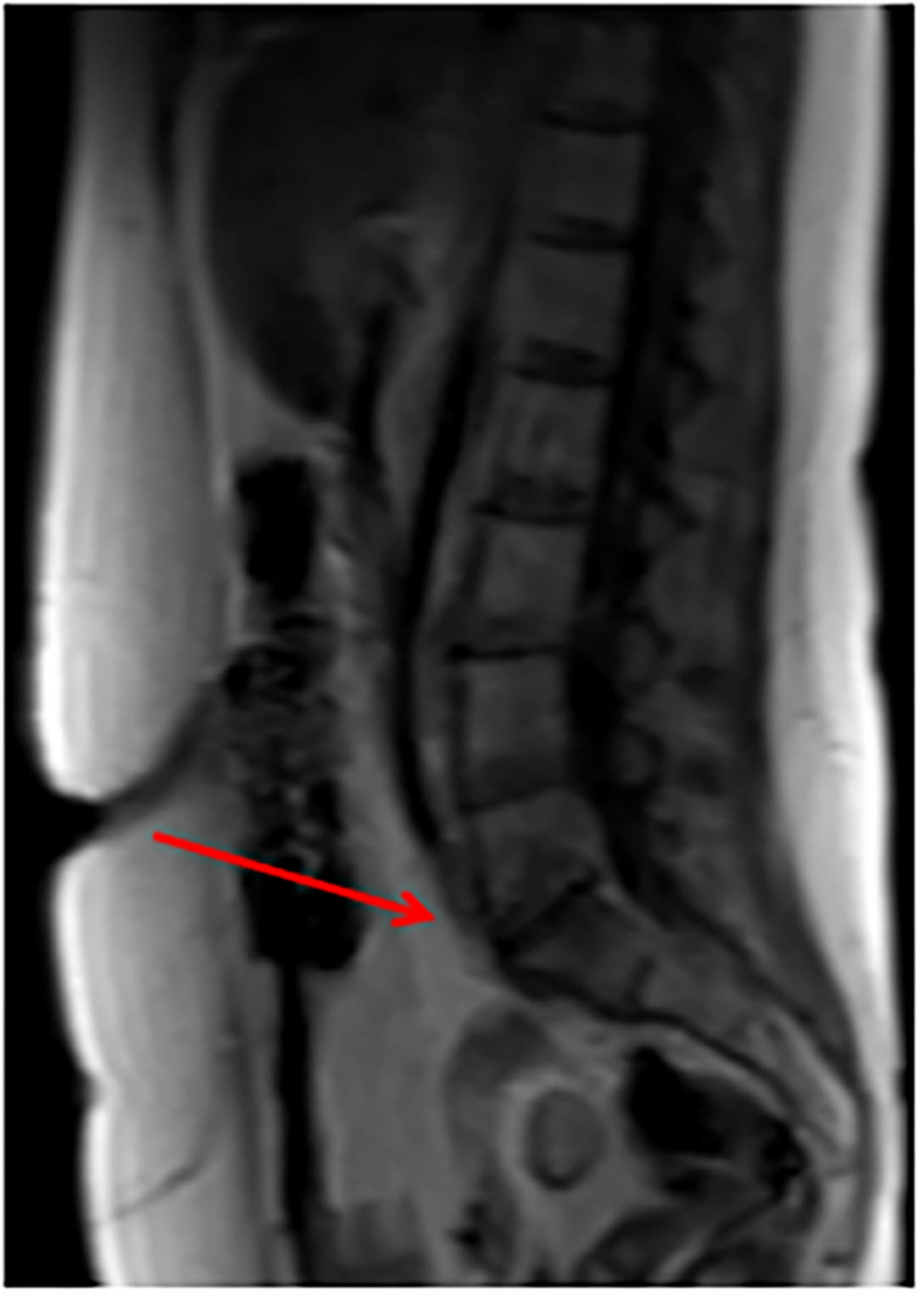
Pelvic MRI 169 days after the second surgery showing a progressive decrease of the purulent collection and a spontaneous resolution of the fistula.

A 51-years-old woman with three previous vaginal deliveries suffered from stage IV uterine and bladder prolapse for 1 year and it worsened in the last 6 months. In May 2020, at the Gynecology Department of the Institute for Maternal and Child Health “IRCCS Burlo Garofolo” of Trieste, the patient underwent laparoscopic hysterectomy; adnexectomy and sacrocolpopexy were performed using a polypropylene mesh anchored with tacks. The postoperative course was initially uneventful and the patient was then discharged on the third postoperative day. One week later, she started to suffer from pelvic pain and approached the emergency department. The transvaginal gynecological ultrasound performed showed a rectovaginal hematic effusion of 80 cc. The woman required hospitalization. Her blood exams were normal except for leukocytosis. She was afebrile and received an intravenous empirical antibiotic treatment with Gentamicin 5 mg/Kg/die and Clindamycin 600 mg x3/die, pending the outcome of blood cultures, which later turned out to be negative. Blood routine examination was normal. After 7 days of antibiotic therapy, the pelvic pain disappeared; the woman became asymptomatic and was discharged. Eighty-three days after the laparoscopic sacrocolpopexy, the patient manifested painful symptoms again and she returned to the emergency room complaining of pelvic pain, back pain, and lumbosciatalgia. A lumbosacral magnetic resonance was performed. The exam showed a signal alteration in the L5-S1 vertebrae with a marked edema of the perivertebral tissues. The patient was therefore hospitalized for the third time at the Gynecology department. Laboratory findings included a white blood cell count of 10,920/ml and C-reactive protein of 54 mg/l. The blood cultures performed resulted in negative again. A magnetic resonance of the pelvis was carried out to complete the study of the lumbosacral district: it confirmed an inflammation of L5-S1 vertebrae, suggesting a spondylodiscitis (Figure 1). Intravenous antibiotic therapy with clindamycin 600 mgx3/die and Gentamicin 5 mg/kg/die started on hospitalization and ended after 14 days. After a multidisciplinary discussion, the medical staff decided that the situation required a revision surgery. Therefore, the patient underwent a second operation 91 days after the laparoscopic sacrocolpopexy. Mesh removal with two metallic tacks, debridement, and drainage of a purulent collection were performed (Figure 2). Furthermore, in those same days, the patient experienced foul-smelling vaginal discharge and had a fever. The intraoperative microbiological samples showed the presence of several colonies of Staphylococcus Aureus resistente alla meticillina (MRSA) and Peptostreptococcus asaccharolyticus. An intravenous targeted antibiotic therapy with vancomycin 2 g/die and rifampicin 600 mg/die was started. Three days later, the patient underwent a CT scan that showed a voluminous collection of pus in the abdominal cavity, concentrated in particular in the pelvis between the vagina and the rectum. Moreover, the CT scan showed a fistula with a median length of about 4 centimeters between the pelvic purulent collection and the vaginal vault (Figure 3). The patient was discharged 21 days after the reoperation. Oral antibiotics (sulfamethoxazole/trimethoprim 160/800 mg cp, 2 cp ×2/die and rifampicin 600 mg/die) were administered to treat the spondylodiscitis for six more weeks. At discharge, the patient was asymptomatic. She underwent a further magnetic resonance performed 121 days after the laparoscopic sacrocolpopexy: the exam showed a volumetric decrease of the pus collection in the pelvis and a persistence of the fistula reaching the vaginal vault. The woman had no pelvic pain, back pain, lumbosciatalgia, or any other symptoms (Table 1). The patient received a close follow-up at our center. Gynecological visits and pelvic ultrasounds were performed monthly. Six months after the second surgical procedure, the woman reported feeling well and denied having any symptoms. Two more pelvic magnetic resonances were performed, respectively, 92 and 169 days after the second surgery, showing a progressive decrease of the purulent collection and a spontaneous resolution of the fistula (Figure 4).

**Table 1:** Data of present case.

**Author**	**Age**	**Initial procedure**	**Time to compli-cation**	**Treatment**	**Fever**	**Symptoms**	**Diagnostic tools indicating spondylodiscitis**	**Possible causes**
Present case	51	LPS sacrocolpopexy	120	Mesh removal, debridement and drainage of a purulent collection; AB	Yes	LBP, pain radiating into the legs, vaginal discharge	MRI	Mesh infection

*LPS, Laparoscopy; AB, Antibiotics; MRI, Magnetic Resonance Imaging*.

## Methods

This retrospective observational descriptive study was approved by our Institutional review board (IRB-Burlo RC 08/2020).

### Eligibility Criteria

We performed a comprehensive search from the electronic databases MEDLINE (Pubmed), Scopus, Web of Science, Embase, CINAHL, and Google Scholar from 1990 to February 2021 in order to identify case reports or case series reporting on spondylodiscitis after rectopexy or sacrocolpopexy. We examined in literature the mean age of patients, the surgical history, the time from initial surgery to spondylodiscitis, the presenting symptoms, the medical and surgical treatment, the diagnostic tools, the type of mesh used, the surgical access, and the possible causes of spondylodiscitis.

### Information Sources

Pubmed, Scopus, Web of Science, Embase, CINAHL, and Google Scholar were searched up to February 2021. The manuscripts considered were published in 1990. Only articles in English were included in the search. The research strategy adopted included different combinations of the following terms: (spondylodiscitis) AND (colpopexy or rectopexy) AND (prolapse). We identified 16 manuscripts from Pubmed database, 64 from Scopus database, and 187 manuscripts from Google Scholar database.

### Study Selection

All studies identified were listed by title, authors, and year of publication. We have followed the PRISMA checklist. The PRISMA flow diagram of the selection process is provided in Table 2. Two independent investigators screened the title and abstracts based on the predefined eligibility criteria. The same two authors reviewed independently the full text of papers identifying those to be included in the review. Discrepancies were resolved by consensus. Thirty-four manuscripts were excluded for duplication. Two-hundred-eight works were excluded for selection criteria. Eighteen manuscripts were detected through the references of the works that had been identified with the research on MEDLINE (PubMed), Scopus, Web of Science, Embase, CINAHL, and Google Scholar.

**Table 2:** Study design.

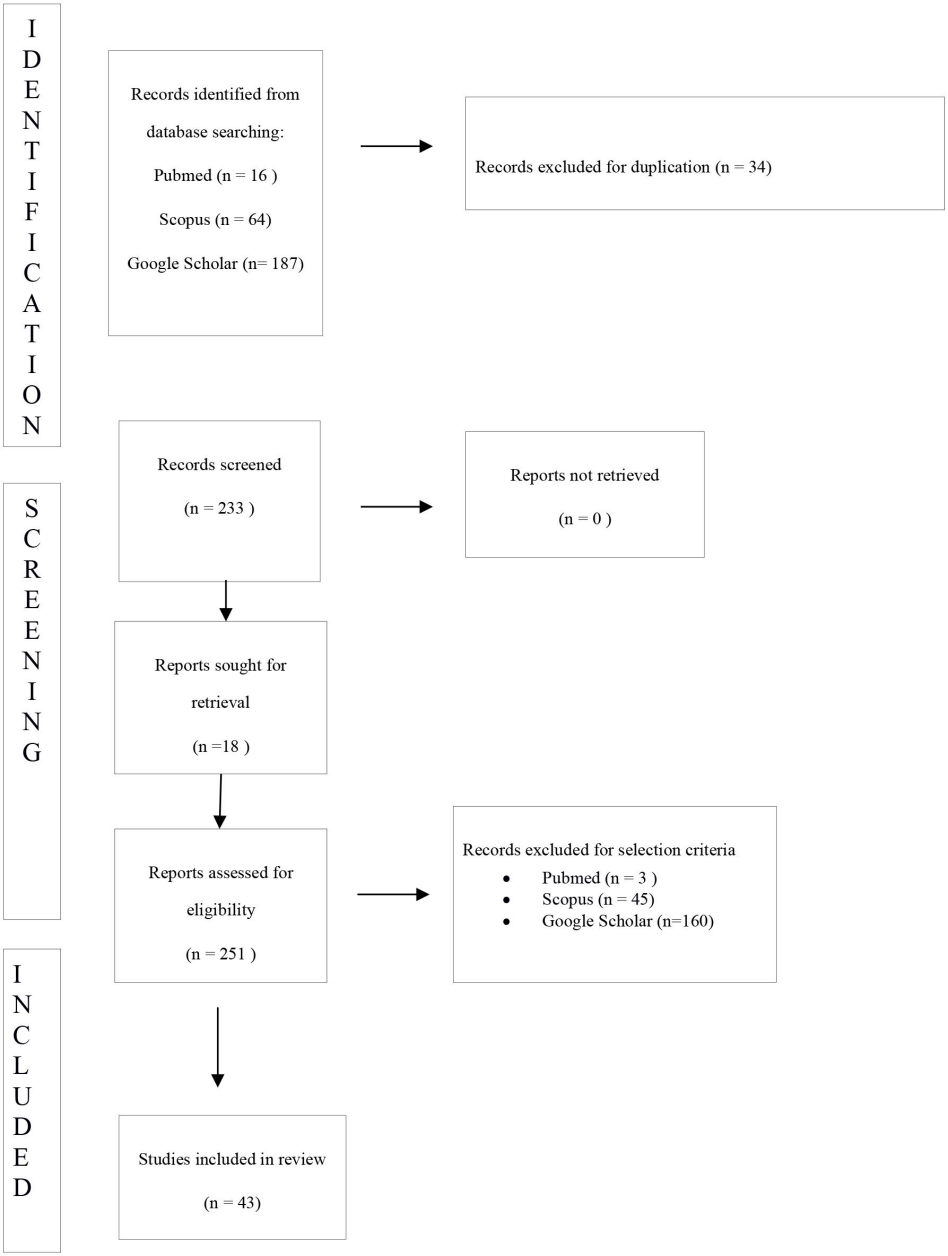

### Assessment of Methodological Quality

The methodological quality of the included studies was assessed using the Joanna Briggs Institute (JBI) Critical Appraisal Checklist for case reports and case series (Supplementary Material).

### Assessment of Risk of Bias

The main risk of bias of the presented work is that almost all papers selected in the literature are case reports.

### Data Analysis

Studies included are almost all case reports due to the rarity of this evenience. For this reason, we presented data in a descriptive manner.

## Results

We describe our clinical case, and then we perform a literature review with MEDLINE (PubMed), Scopus, Web of Science, Embase, CINAHL, and Google Scholar.

We found 41 manuscripts about spondylodiscitis following sacral colpopexy or rectopexy or combined sacro/rectopexy and two reports of spondylodiscitis following a sacrohysteropexy. Summary of the characteristics is presented in Table 3. Fifty-two women with a median age of 59.6 years were diagnosed with spondylodiscitis after a median of 332 days from the initial surgery. Initial surgery consisted of sacrocolpopexy (*n* = 42; 80.8%), rectopexy (*n* = 6; 11.5%), hysteropexy (*n* = 2; 3.8%), and combined sacrocolporectopexy (*n* = 2; 3.8%). The most common surgery technique used was laparoscopic access (*n* =27; 51.9%), followed by open access in 34.6% of cases (*n* = 18), and robotic access in the minority of cases (*n* = 7; 13.4%). Pexy was performed using synthetic meshes (*n* = 38; 73.1%), biologic meshes (*n* = 3; 5.8%), in four cases (7.7%), the type of mesh used was not specified. Direct sutures were used in five cases (9.6%) and the type of technique used was not specified in two cases (3.8%). All patients complained of back pain (*n* = 50; in two cases, the symptoms were not specified), almost half of the patients (42.3%, *n* = 22) had fever. Other common presenting symptoms were pain radiation into the legs (*n* = 17; 32.7%) and vaginal discharge (*n* = 5; 9.6%). A mesh erosion (*n* = 9; 17.3%) or a fistula formation (*n* = 8; 15.4%) was detected in a minority of cases. The gold standard for diagnosis of postoperative spondylodiscitis is pelvic magnetic resonance. Most of the cases analyzed by our review have been diagnosed performing a pelvic resonance (*n* = 40; 76.9%), in one case, the diagnosis was reached using a CT scan. Six women underwent both exams, while in five patients, the diagnostic tool chosen was not specified (Tables 3, 4). Antibiotics alone were effective in only 14 cases (26.9%), whereas 67.3% of the patients (*n* = 35) had to undergo additional surgical treatment.

**Table 3:** Studies included in the systematic review listed in alphabetic order.

**References**	**Case**	**Age**	**Initial procedure**	**Time to compli-cation**	**Treatment**	**Fever**	**Symptoms**	**Diagnostic tools indicating spondylodiscitis**	**Possible causes**
Anand et al. (3)	1	70	Robotic supracervical hysterectomy with sacrocolpopexy	90	Mesh removal + laminectomy and anterior discectomy; AB	No	LBP, radiating leg pain for 3 months	CT/MRI	Recurrent UTI
Api et al. (4)	2	53	Total hysterectomy with sacral colpopexy via LPS	6	Mesh removal; AB	Yes	LBP, radiating pain to the upper tights for 6 days	MRI	NS
	3	65	Via LPT	53	Mesh removal, AB	Yes	LBP, radiating pain to the upper tights for 6 days	MRI	NS
Apostolis et al. (5)	4	66	LPS supracervical hysterectomy and sacrocolpopexy	10	Laminectomy and debridment of epidural flegmon; AB	Yes	LBP for one and a half week	MRI	Past history of dental extraction of infected teeth
Arsene at al. (6)	5	NS	Hysterectomy, LPT sacrocolpopexy	30	Mesh removal; AB	Yes	LBP, vaginal discharge	NS	NS
Belooseky at al. (7)	6	74	LPT sacrocolpopexy	50	L5 laminectomy	Yes	LBP for 7 weeks	CT/MRI	UTI
Boyd et al. (8)	7	71	Robotic sacrocolpopexy	42	LPS mesh removal; AB	Yes	LBP for 6 weeks	CT/MRI	Suture placement 2 cm above the sacral promontory, sacral then the usual level of placement
Brito et al. (9)	8	61	Subtotal LPS hysterectomy and sacrocolpopexy	12	Mesh removal; AB	Yes	LBP for 12 days	MRI	NS; Breast cancer
Cailleux et al. (10)	9	54	LPT supracervical hysterectomy and sacral colpopexy	66	Mesh removal; AB	Yes	LBP for 1.5 months	MRI	Postoperative pelvic abscess
	10	41	Hysterectomy and LPT sacralcolpopexy	91	Only AB	Yes	LBP for 4 months, vaginal discharge	MRI	NS
	11	55	Hysterectomy and LPT sacralcolpopexy	66	Only AB	Yes	LBP for 3 months	MRI	NS
	12	56	Hysterectomy and LPT sacralcolpopexy	115	Only AB	Yes	LBP for 4 months, vaginal discharge	MRI	NS
	13	59	Hysterectomy and LPT sacralcolpopexy	76	Only AB	Yes	LBP for 6 months	MRI	NS
Collins et al. (11)	14	74	LPT sacral colpopexy	2,920	IVC filter, mesh removal and abscess debridement; AB	No	LBP for 8 years	MRI	UTI
Cosson et al. (12)	15	45	LPS sacropexy	730	Mesh removal; AB	No	LBP for 2 years	MRI	UTI
Cranney et al. (13)	16	72	LPT sacral colpopexy	30	Mesh removal, discectomy, spinal fusion; AB	Yes	LBP for 4 weeks	MRI	UTI
Dalwai et al. (14)	17	NS	LPS sacrocolpopexy	7	NS	NS	LBP for 1 week	MRI	Inadvertent placement of the sacrocolpopexy screw into the lumbar intervertebral disk space at L5–S1
	18	NS	LPS sacrocolpopexy	7	NS		LBP for 1 week	MRI	Inadvertent placement of the sacrocolpopexy screw into the lumbar intervertebral disk space at L5–S1
Descargues et al. (15)	19	54	LPS hysterectomy, sacrocolpopexy, salpingectomy	540	Only AB	Yes	LBP, Radiculopatia L5-S1	MRI	Mesh erosion
Downing et al. (16)	20	52	LPS uterus-preserving cervicosacropexy	420	Abdominal hysterectomy, salpingo-oophorectomy, mesh-removal; AB	No	LBP radiating to the hip and leg for 14 months	MRI	Vaginal mesh erosion
Draaisma et al. (17)	21	45	LPS sacral ventral rectopexy	30	Mesh removal, deviating colostomy; AB	Yes	LBP radiating to both legs for 1 month	MRI	Not noted. Systemic lupus erithematosus and hydroxychloroquine
	22	55	LPS sacral ventral rectopexy		Only AB	Yes	LBP for 2 months	MRI	NS
Durdag et al. (18)	23	NS	LPS hysterectomy and sacrocolpopexy	90	LPS mesh removal, anterior L5 – S1 discectomy; AB	No	LBP for 3 months	MRI	NS
Feng et al. (19)	24	64	Robotic hysterectomy, sacrocolpopexy, urethral sling	30	Mesh removal; AB		LBP	CT/MRI	NS
Grimes et al. (20)	25	63	Robotic – assisted sacral colpopexy	120	Mesh removal and debridement of the infected area. Exposure of the posterior spine with screw placement. Anterior L4-L5 discectomies and corpectomies. Posterior iliac screws and spine fusion; AB	No	LBP, radiating pain to the buttock for 4 months	CT/MRI	Yeast vaginitis
Hart et al. (21)	26	42	Total abdominal hysterectomy, LPT sacral colpopexy	150	Transvaginal removal, LPT, sacral debridement, partial vaginectomy; AB	Yes	LBP weakness in the lower extremities for 5 months	MRI	Vaginal mesh erosion
Jallad et al. (22)	27	NS	LPS sacrocolpopexy and ventral rectopexy	30	Excision of the sacral portion of the graft; AB	NS	LBP	NS	NS
Jenson et al. (23)	28	67	LPS sacral colpopexy	120	LPS mesh removal; AB	No	LBP for 4 months	MRI	NS
Kapoor et al. (24)	29	63	LPS sacrocolpopexy	21	Only ABs	Yes	LBP for 3 weeks	MRI	Wound infection at one of the port sites
Kumara et al. (25)	30	32	LPS sacrohysteropexy	15	LPT; AB	Yes	LBP radiating to the buttocks and left lower limb	MRI	Mesh and fasteners infection
Miksic et al. (26)	31	81	LPS sacrocolpopexy	126	Only AB	no	LBP, pain radiating into the right leg	MRI	Iatrogenic anaerobic spinal epidural abscess with sacral spondylodiscitis caused by direct extension of bacteria through anchoring material in the sacrum Old, frial, patient
Muffy et al. (27)	32	46	Transvaginal mesh followed by robot assisted sacral colpopexy	180	LPT mesh removal, discectomy; AB	No	LBP for 1 year	MRI	Vaginitis Diabetes mellitus
Muller et al. (2)	33	60	LPS proximal rectopexy	63	Fistula resection, resection of the anastommosis, fashioning of a new anastomosis and a protective loop ileostomy; AB	No	LBP, pain radiating in both legs	MRI	Fistula from dorsal rectopexy
Nosseir et al. (28)	34	55	Robotic hysterectomy, sacrocolpopexy, transobturathor, suburethral sling	70	Only AB	No	LBP for 6 weeks	MRI	NS
Nunez-Pereira et al. (29)	35	80	LPT sacrocolpopexy	2520	Abscess debridement, lumbar fusion -L1-L4 decompression -mesh removal, rectosigmoidal resection, protective loop ileostomy; AB	Yes	LBP, radiating leg pain for 7 years, vaginal discharge	MRI	Rectal fistula following mesh penetration
Pasquer et al. (30)	36	76	LPS rectopexy and cistopexy	30	Harmann's procedure; AB	Yes	LBP	MRI/CT	NS
Probst et al. (31)	37	81	LPS resection rectopexy	90	Only AB	No	LBP and pain radiating to both legs	MRI	Presacral seroma/Pseudomonas sepsis
Propst et al. (32)	38	66	Robotic assisted LPS sacral colpopexy, ventral rectopexy	60	Laminectomy; discectomy, mesh removal; AB	No	LBP, radiating bilateral leg pain for 8 weeks	MRI	The location of the mesh was above the S1 vertebra and not within the disc space
	39	55	Total abdominal hysterectomy and LPT sacral colpopexy	1095	Mesh removal; surgical mesh debridement; AB	No	LBP, limited mobility for 3 years	CT	Mesh erosion at the vaginal apex
Qu et al. (33)	40	46	LPS sacrohysteropexy	30	LPS mesh removal and hysterectomy. 5 months later: debridement and laminectomy; AB	No	LBP, pain between the right iliac crest and the buttock, pain in the right lower limb	MRI	Mesh suture placed higher than it usual level
Rajamahesvary et al. (34)	41	42	Abdominal hysterectomy and LPT sacrocolpopexy; AB	42	Mesh removal, AB	No	LBP restricting physical movements and ambulation for 8 weeks	MRI	Mesh erosion
Rivoire et al. (35)	42	NS	LPS sacrocolpopexy	Not specified	NS	NS	NS	NS	Patient had diabetes
Roth et al. (36)	43	76	LPS sacral colpopexy	2795	LPS enterolysis, drainage of the abscess, and expalantation of the remaning mesh; AB	No	LBP, vaginal discharge	NS	Mesh erosion Pelvic abscess over the sacrum Colovaginal fistula
Salman et al. (37)	44	59	LPT Sacrocolpopexy	120	Abscess debridement, posterior stabilization; AB	No	LBP radiating to both legs for 4 months	MRI	NS
Sergent et al. (38)	45	NS	LPS sacrocolpopexy	120	Only AB	NS	NS	NS	NS
Taylor et al. (39)	46	64	LPS assisted vaginal hysterectomy, sacral colpopexy	465	Mesh removal, laminectomy; AB	No	LBP for 14 months	MRI	Vaginal mesh erosion
Tymchak et al. (40)	47	61	Transvaginal hysterectomy with LPT sacrocolpopexy	60	Abdominal mesh removal; AB	No	LBP for 1 month, L5 radiculopathy	MRI	NS
Ugurlucan et al. (41)	48	52	Total LPS hysterectomy and sacrocolpopexy	21	LPS mesh excition; AB	No	LBP for 3 weeks	MRI	NS
Voelker et al. (42)	49	58	LPS sacral colpopexy	1095	Removal of the neovagina, debridement, excision of the intervetebral disk with bone graft replacement, dorsal instrumentation of the segments L5-S1; AB	Yes	LBP for 3 years	MRI	NS Malignant melanoma of the vagina
Vujovic et al. (43)	50	50	LPS ventral mesh, rectopexy; AB	42	Surgical screw removal; AB	No	LBP for 11 weeks	MRI	NS
Weidner et al. (44)	51	67	LPTsacral colpopexy	1,825	Only AB	No	LBP for 5 years	MRI	NS
	52	56	Total abdominal hysterectomy, LPT sacral colpopexy	120	Only AB	No	LBP for 4 months	MRI	NS

*LPS, Laparoscopy; AB, Antibiotics; MRI, Magnetic Resonance Imaging; CT, Computed tomography; UTI, Urinary tract Infection; LPT, Laparotomy; LBP, Low Back Pain; NT, Not Specified*.

**Table 4:** Baseline characteristics, presenting symptoms and type of treatment (*n* = 52).

Mean age (average)	59.6
Gender (male:female)	0:52
Initial surgery (average)	
Colpopexy	42 (80.8%)
Rectopexy	6 (11.5%)
Hysteropexy	2 (3.8%)
Combined sacrocolporectopexy	2 (3.8%)
Time to complication (days; average)	332
Sign and symptoms (average)
Back pain	50 (96.1%)
Fever	22 (42.3%)
Pain radiating into the legs	17 (32.7%)
Vaginal discharge	5 (9.6%)
Not specified	2 (3.8%)
Access (average)
Laparoscopic	27 (51.9%)
Robotic	7 (13.4%)
Open	18 (34.6%)
Fixation technique (average)
Mesh	45 (86.6%)
• Syntetic	• 38 (73.1%)
• Biological	• 3 (5.8%)
• Not specified	• 4 (7.7%)
Non-absorbable direct suture	5 (9.6%)
Not specified	2 (3.8%)
Mesh erosion (average)	9 (17.3%)
Vaginal mesh erosion (average)	8 (15.4%)
Rectal mesh erosion (average)	1 (1.9%)
Fistula (average)	9 (17.3%)
Diagnostic tool (average)
• RM	40 (76.9%)
• TC	1 (1.9%)
• RM/TC	6 (11.5%)
• Not specified	5 (9.6%)
Reoperation/antibiotics (average)	35 (67.3%)
Antibiotics alone (average)	14 (26.9%)
Not specifed (average)	3 (5.8%)

## Discussions

Surgeons should be aware of the potential risk of spondylodiscitis caused by a sacrocolpopexy and (or) rectopexy with and without the use of a mesh. Sacrocolpopexy is described to be one of the safest procedures for the surgical treatment of prolapse (6). Monofilament polypropylene mesh is the graft of choice (45). In literature, there are 52 cases of lumbar spondylodiscitis as a result of sacrocolpopexy and (or) rectopexy or sacrohysteropexy performed using synthetic meshes (*n* = 38), biologic meshes (*n* = 3), direct sutures (*n* = 5), with four cases where the type of mesh used is not specified and two cases where the technique is not mentioned (6). The characteristics of patients are summarized in Tables 3, 4. The mesh is placed on the ventral side of the vagina and fixated with stitches or tacks on the sacral promontory (1). Qu et al. reported that the possible causes of spondylodiscitis are mainly related to the mesh (32%) and to other infections (29%), including urinary tract infections, vaginitis, postoperative pelvic abscess, wound infection, dental extraction of infected teeth in one case (33), while the other causes of spondylodiscitis are not known. In their manuscript, mesh-related causes of spondylodiscitis include vaginal mesh erosion, mesh penetration into the rectum (one case), and suture placement on the sacral anterior ligament at a higher level than the usual fixation (33). Mesh erosion after ventral rectopexy and sacropexy varies greatly across studies and are reported rates between 1.3 and 6%. The deterioration of the mesh may predispose to infections, leading to the migration of bacteria from the vagina or rectus to the prothesis and its fixation site (46). Sacral colpopexy can be performed with open, laparoscopic, or robotic– assisted techniques. Our research revealed that 51.9% of cases underwent a laparoscopic colpopexy. An open access was adopted in 18 cases (34.6%) and a robotic-assisted surgery was performed in a minority of patients (seven cases; 13.4%). This may suggest laparoscopy as a risk factor for spondylodiscitis. Nevertheless, it should be taken into account that laparoscopy is the preferred technique for this type of surgery (45).

Interestingly, the study by Unger et al. compared the results between laparoscopic sacrocolpopexy and robotic-assisted sacrocolpopexy in 406 women. The rate of postoperative osteomyelitis was similar between the two groups (47). Grevez et al. reported the absence of postoperative spondylodiscitis among the 20 cases of abdominal promontofixation analyzed in their systematic review (48). The different haptic feedback of the three surgical techniques could be the key to explain these data. A decreased haptic feedback could elevate the risk of penetration deeper into the anterior longitudinal ligament, which allows bacteria to directly access the bone or disc. There are insufficient and conflicting data about the possible risk associated with performing hysterectomy (total or subtotal) or uterus preservation during sacrocolpopexy. The issue of uterine preservation or excision during the procedure requires further clarification (45). The most common type of mesh used is the polypropylene (45). From our data emerges that 73.1% of postoperative spondylodiscitis arises after a surgical prolapse correction with a synthetic prosthesis. It can be hypothesized that synthetic grafts can be a vehicle for germs colonization and their subsequent spreading into the disc and the bone. However, a bias could be represented by the almost exclusive use of this type of synthetic grafts in all sacrocolpopexy. So, we cannot generalize considering them as a risk factor for spondylodiscitis. It is known that the origin of spondylodiscitis is multifactorial and it can occur with classic sutures (6). The mesh can be anchored using stitches of different types (also barbed one) (49) or tacks. It could be supposed that tacks could penetrate more easily into the anterior longitudinal ligament exceeding its thickness and leading to spondylodiscitis. The majority of articles do not describe the way of mesh fixation to the anterior longitudinal ligament. The lack of data does not allow to have certain information about this aspect and to understand which is the best tool of fixation. However, surgeons are able to minimize the risk of spondylodiscitis by carefully placing the presacral fixation, putting stitches or tacks into the anterior longitudinal ligament avoiding the disc space (49). The surgeon has to keep in mind that the anterior longitudinal ligament is only 1–2 mm thick and this could lead to an easy perforation of it (50). Furthermore, mesh suture load into the vaginal wall should be minimized in order to decrease the risk of organism migration between the mucocutaneous layer and the mesh (51). From our review emerged that the onset of spondylodiscitis varies greatly, from 1 month to 8 years after surgery. In 76.9% of cases (40 cases), spondylodiscitis occurred within 1 year after surgery. The mean time of presentation of this postoperative complication is 332 days. This could be explained by fewer painful symptoms, which delay the diagnosis of complications. All patients complained of back pain (*n* = 50; in two cases, symptoms were not specified), pain radiating into the legs, and consecutive motor weakness and sensory changes are only found in a minority (*n* = 17; 35%). Less than half of the patients (*n* = 22; 42.3%) have fever. Some women declare also vaginal discharge (*n* = 5; 9.6%). Pelvic magnetic resonance appears to be the gold standard for the diagnosis of spondylodiscitis. It is the diagnostic tool used in the majority of clinical cases (*n* = 40; 76.9%). The magnetic resonance demonstrates to be the most sensitive (93–96%) and specific (92–97%) imaging modality for the diagnosis of spondylodiscitis. On the other hand, CT gives a more detailed image of bone destruction (second choice) (20). In the presence of typical clinical symptoms, imaging studies of the lower spinal cord should be performed without delay. The diagnostic process should require blood and urine cultures completed by a gynecological evaluation to exclude vaginal infections (20). From the review of Mavrogenis et al., it emerges that Staphylococcus aureus has become the most frequent bacterium responsible for vertebral infections, accounting for 20–84% of all cases (52). Additionally, Enterobacteriaceae spp. are implicated in 7–33% of pyogenic vertebral infections. Escherichia coli is the most common pathogen in this group, followed by Proteus and Klebsiella. Streptococci and Enterococci are common causes responsible for 5% to 20% of cases, whereas, anaerobes are isolated in <4% (20, 52–56). When blood cultures are negative, CT-guided biopsy is recommended (20). Our comprehensive research revealed that in many cases, the conservative treatment with antibiotics is not enough and surgical therapy is needed in 67.3% of the cases (Table 4). A possible explanation could be that prosthetic material acts as an infection route and reservoir for bacteria, as reported by Muller et al. and Qu et al. in their reviews. Surgical treatment usually includes mesh removal, laminectomy, discectomy, and spine-stabilizing procedures (these last in case of either nerve compression or spinal instability) (2, 33). Intravenous antibiotic therapy is recommended for 4–8 weeks; after that, a 3-month course of oral antibiotic therapy should follow (2).

The strength of our study is the long period of time overviewed in literature: we analyzed the cases of postoperative spondylodiscitis arised in the last 30 years. All the studies selected during the eligibility phase (according to the PRISMA guidelines) have been further evaluated by manual comparison of populations, study settings, and authors to exclude overlapping cases. However, the limitation of our study is the retrospective nature of it, and the main risk of bias is represented by the presence of almost all case reports among the papers selected.

Although spondylodiscitis remains a rare evenience, it can lead to irreversible complications. Indications to surgical treatment include doubtful diagnosis, progressive neurological deficits, progressive spinal deformity, failure to respond to treatment, and unresolved pain. Today, the time spanning from the initial procedure to the diagnosis of spondylodiscitis varies greatly and ranges from 6 days to 8 years. A reasonable level of suspicion and a certain degree of multidisciplinary approach are fundamental for a prompt diagnosis and a successful treatment.

## Data Availability Statement

The original contributions presented in the study are included in the article/Supplementary Material, further inquiries can be directed to the corresponding author/s.

## Ethics Statement

Written informed consent was obtained from the relevant individual for the publication of any potentially identifiable images or data included in this article.

## Author Contributions

GS and GT: conceptualization and writing—original draft preparation. GS, GT, and GR: methodology. GT and AL: software. GS, FR, and GR: validation. GT and FR: formal analysis. GS, FM, and GD: investigation. GS, GD, FM, and GR: data curation. GS, FR, AL, and GT: writing—review and editing. AL: visualization. FR and GR: supervision. FM and GD: project administration. All authors have read and agreed to the published version of the manuscript.

## Funding

This research was supported by a grant from the Institute for Maternal and Child Health IRCCS Burlo Garofolo (RC 08/2020).

## Conflict of Interest

The authors declare that the research was conducted in the absence of any commercial or financial relationships that could be construed as a potential conflict of interest.

## Publisher's Note

All claims expressed in this article are solely those of the authors and do not necessarily represent those of their affiliated organizations, or those of the publisher, the editors and the reviewers. Any product that may be evaluated in this article, or claim that may be made by its manufacturer, is not guaranteed or endorsed by the publisher.
