# Supplementary material for: Spondylodiscitis After Surgery for Pelvic Organ Prolapse: Description of a Rare Complication and Systematic Review of the Literature
Source: Front Surg. 2021 Oct 29;8:741311. doi: 10.3389/fsurg.2021.741311 (PMC8586200; doi:10.3389/fsurg.2021.741311)
Supplement: Supplementary file 1 [file Table_1.DOCX]

			JBI Critical Appraisal Checklist for Case Reports					
**Articles**	**D1**	**D2**	**D3**	**D4**	**D5**	**D6**	**D7**	**D8**
Anand M. et al.	Yes	Yes	Yes	Yes	Yes	Yes	Yes	Yes
Api M. et al.	Yes	Yes	Yes	Yes	Yes	No	Yes	Yes
Apostolis C.A. et al.	Yes	Yes	Yes	Yes	Yes	Yes	Yes	Yes
Arsene E. et al.	Unclear	Unclear	No	No	Unclear	Unclear	Unclear	Yes
Beloosesky Y. et al.	Yes	Yes	Yes	Yes	Yes	Yes	Yes	Yes
Boyd B. et al.	Yes	Yes	Yes	Yes	Yes	Yes	Yes	Yes
Brito L.G. et al.	Yes	Yes	Yes	Yes	Yes	Yes	Yes	Yes
Cailleux N. et al.	unclear	Yes	Yes	Yes	Yes	Unclear	Yes	Yes
Collins S.A. et al.	Yes	Yes	yes	Yes	Yes	Yes	Yes	Yes
Cosson M. et al.	Yes	Yes	Yes	Yes	yes	Yes	Yes	Yes
Cranney A. et al.	Yes	Yes	Yes	Unclear	Unclear	Unclear	Unclear	Yes
Dalway R. et al.	Unclear	Yes	Unclear	No	No	No	No	No
Descargues G. et al.	Yes	Yes	Yes	Yes	Yes	Yes	Yes	Yes
Downing K.T. et al.	Unclear	Yes	Yes	Yes	yes	yes	yes	Yes
Draaisma W.A. et al.	Yes	Yes	Yes	Yes	Yes	Unclear	Yes	Yes
Durdag G.D. et al.	Unclear	Yes	Yes	Yes	Yes	Yes	Yes	Yes
Feng T.S. et al.	Yes	Yes	Yes	Yes	Yes	Yes	Yes	Yes
Grimes C.L. et al.	Yes	Yes	Yes	Yes	Yes	Yes	Yes	Yes
Hart S.R. et al.	Yes	Yes	Yes	Yes	Yes	Yes	Yes	Yes
Jallad C. et al.	No	Unclear	Unclear	Unclear	Unclear	Unclear	Unclear	Yes
Jenson A.V. et al.	Yes	Yes	Yes	Yes	Yes	Yes	Yes	Yes
Kapoor B.T. et al.	Yes	Yes	Yes	Yes	Yes	Unclear	Yes	Yes
Kumara D.M.A. et al.	Yes	Yes	Yes	Yes	Yes	Yes	Yes	Yes
Miksic N.G. et al.	Yes	Yes	Yes	Yes	yes	Yes	Yes	Yes
Muffy T.M. et al.	Yes	Yes	Yes	Yes	Yes	Yes	Yes	Yes
Muller P.C. et al.	Yes	Yes	Yes	Yes	Yes	Yes	Yes	Yes
Nosseir S.B. et al.	Unclear	Yes	Yes	Yes	Yes	Yes	Yes	Yes
Núñez-Pereira . et al.	Yes	Yes	Yes	Yes	Yes	Yes	Yes	Yes
Pasquesr A. et al.	Yes	Yes	Yes	Yes	Yes	Yes	Yes	Yes
Probst P. et al.	Unclear	Yes	Yes	Yes	Yes	Unclear	Yes	Yes
Propst K. et al.	Unclear	Yes	Yes	Yes	Yes	Yes	Yes	Yes
Qu D.C. et al.	Yes	Yes	Yes	Yes	Yes	Yes	Yes	Yes
Rajamaheswary N. et al.	Yes	Yes	Yes	Yes	Yes	Yes	Yes	Yes
Rivoire C. et al.	Yes	Yes	Yes	Yes	Yes	Yes	Yes	Yes
Roth T.M. et al.	Yes	Yes	Yes	Yes	Yes	Unclear	Yes	Yes
Salman M.M. et al.	Yes	Yes	Yes	Yes	Yes	Yes	Yes	Yes
Sergent F. et al.	Unclear	Unclear	Unclear	No	unclear	No	No	Yes
Taylor G.B. et al.	Yes	Yes	Yes	Yes	Yes	Yes	Yes	Yes
Tymchak Z.A. et al.	Yes	Yes	Yes	Yes	Yes	Yes	Yes	No
Ugurlucan F.G. et al.	Yes	Yes	Yes	Yes	Yes	Yes	Yes	Yes
Volker A- et al.	Yes	Yes	Yes	Yes	Yes	Yes	Yes	Yes
Vujovic Z. et al.	Yes	Yes	Yes	Yes	Yes	Yes	Yes	Unclear
Weidner A.C et al.	Yes	Yes	Yes	Yes	Yes	Yes	Yes	Yes

**D1**	Where patient's demographic characteristics clearly described?
**D2**	Was the patient's history clearly described and presented as a timeline?
**D3**	Was the current clinical condition of the patient on presentation clearly described?
**D4**	Were diagnostic tests or assessemet methods and the results clearly described?
**D5**	Was the intervention(s) or treatment procedure(s) clearly described?
**D6**	Was the post-intervention clinical condition clearly described?
**D7**	Were adverse events (harms) or unticipated events identified and described?
**D8**	Does the case report provide takeaway lessons?

			JBI Critical Appraisal Checklist for Case Series							
**Articles**	**Articles**	**Articles**	**Articles**	**Articles**	**Articles**	**Articles**	**Articles**	**Articles**	**Articles**	**Articles**
Cailleux N. et al.	Yes	Yes	Yes	Yes	Yes	Unclear	Yes	Yes	Unclear	Yes
Rivoire C. et al.	Yes	Yes	Yes	Yes	Yes	No	Yes	Yes	Yes	Yes

**D1**	Were there clear criteria for inclusion in the case series?
**D2**	Was the condition measured in a standard, reliable way for all partepipants included in the case series?
**D3**	Were valid methods used for identification of the condition for all partecipants included in the case series?
**D4**	Did the case series have consecutive inclusion of partecipants?
**D5**	Did the case series have complete inclusion of partecipants?
**D6**	Was there clear reporting of the demographics of the partecipants in the study?
**D7**	Was there clear reporting of clinical information of the partecipants?
**D8**	Were the outcomes or follow up results of cases clearly reported?
**D9**	Was there clear reporting of the presenting site(s)/clinic(s) demographic information?
**D10**	Was statistical analysis appropriate?
